# Emergency Politics After Globalization

**DOI:** 10.1093/isr/viab021

**Published:** 2021-06-01

**Authors:** Monika Heupel, Mathias Koenig-Archibugi, Christian Kreuder-Sonnen, Markus Patberg, Astrid Séville, Jens Steffek, Jonathan White

**Affiliations:** Otto Friedrich University Bamberg; The London School of Economics and Political Science; Friedrich Schiller University Jena; University of Hamburg; Ludwig-Maximilians-University Munich; Technical University of Darmstadt; The London School of Economics and Political Science

**Keywords:** emergency politics, global governance, regional governance, European Union, Palabras clave, política de emergencia, gobernanza mundial, gobernanza regional, unión europea, Mots clés, politiques d'urgence, gouvernance mondiale, gouvernance régionale, union européenne

## Abstract

Exceptional times call for exceptional measures—this formula is all too familiar in the domestic setting. Governments have often played loose with their state's constitution in the name of warding off an urgent threat. But after decades of increasing interconnectedness and emerging transnational governance, today one sees new forms of emergency politics that are cross-border in range. From the European Union to the World Health Organization, from supranational institutions to state governments acting in concert, the logic of emergency is embraced in international contexts, with Covid-19 the latest occasion. This Forum offers an entry-point into this emerging phenomenon. Taking as its point of departure two recent books, it examines the origins, forms, effects and normative stakes of emergency politics beyond the state. Among the matters discussed are the concept of emergency politics, the historical context of its contemporary forms, the patterns of decision-making associated with it, the implications for the legitimacy of transnational institutions, and the constitutional and political ways in which it might be contained. Transnational emergency politics seems likely to remain a central feature of the coming years, and our aim is to further its study in international relations.

## Abstract (es)

En épocas excepcionales, se requieren medidas excepcionales, y esta fórmula es demasiado familiar en el marco nacional. Con frecuencia, los gobiernos se han tomado a la ligera la constitución de su estado en nombre de repeler una amenaza urgente. Pero, después de décadas marcadas por el aumento en la interconexión y el surgimiento de gobiernos transnacionales, hoy se ven nuevas formas de política de emergencia que tienen un alcance transfronterizo. Desde la Unión Europea (UE) hasta la Organización Mundial de la Salud (OMS), y desde las instituciones supranacionales hasta los gobiernos estatales que aúnan esfuerzos, la lógica de la emergencia se incluye en los contextos internacionales, siendo el más reciente la pandemia de la COVID-19. Este foro ofrece una vía de acceso a este fenómeno emergente. Tomando como punto de partida dos libros recientes, se examinan los orígenes, las formas, los efectos y los intereses normativos de la política de emergencia más allá del estado. Entre los temas que abarca, se encuentran el concepto de política de emergencia, el contexto histórico de sus formas contemporáneas, los patrones de toma de decisiones que se relacionan con esta, las implicaciones para la legitimidad de las instituciones multinacionales, y las maneras constitucionales y políticas en las que puede incluirse. Parece probable que la política de emergencia transnacional continúe siendo una característica central de los próximos años, y nuestro objetivo es promover su estudio en las relaciones internacionales.

## Abstract (fr)

À temps exceptionnel, mesures exceptionnelles : cette formule n'est que trop familière dans le contexte national. Des gouvernements ont souvent interprété la constitution de leur État comme ils l'entendaient au nom de l’écartement d'une menace urgente. Mais après des décennies d'interconnexion croissante et d’émergence d'une gouvernance transnationale, nous assistons aujourd'hui à de nouvelles formes de politiques d'urgence qui ont une portée transfrontalière. De l'Union Européenne (UE) à l'Organisation Mondiale de la Santé (OMS), des institutions supranationales aux gouvernements étatiques agissant de concert, la logique de l'urgence est adoptée dans des contextes internationaux, le COVID-19 en étant la dernière occasion. Cette tribune propose une approche de ce phénomène émergent. Prenant deux livres récents comme point de départ, elle examine les origines, les formes, les effets et les enjeux normatifs des politiques d'urgence au-delà de l’État. Parmi les questions abordées figurent le concept de politique d'urgence, le contexte historique de ses formes contemporaines, les modèles de prise de décisions qui y sont associés, les implications pour la légitimité des institutions transnationales, et les moyens politiques et constitutionnels par lesquels elle pourrait être endiguée. Les politiques d'urgence transnationales semblent devoir rester un élément central des années à venir, et notre objectif est d'approfondir leur étude en relations internationales.

## Introduction

Christian Kreuder-Sonnen^1^, Jonathan White^2^, Monika Heupel^3^, Mathias Koenig-Archibugi^2^, Markus Patberg^4^, Astrid Séville^5^ and Jens Steffek^6^

^1^Friedrich Schiller University Jena

^2^The London School of Economics and Political Science

^3^Otto Friedrich University Bamberg

^4^University of Hamburg

^5^Ludwig-Maximilians-University Munich

^6^Technical University of Darmstadt

Crises often provoke political reactions that bend or suspend established norms and rules. This phenomenon has found constitutional expression at the domestic level in the “state of exception,” theorized by Carl Schmitt in the 1920s and an enduring concern for legal and political observers today. Liberal democracies around the world seem as vulnerable as ever to suspensions of their core commitments (Honig 2009; Lazar 2009). But contingencies that cross borders, such as the Euro crisis or Covid-19, nowadays also incite emergency politics *beyond* the state. From the European Union (EU) to the World Health Organization (WHO), from supranational institutions to state governments acting in concert, one sees the logic of emergency embraced in international contexts. What are the origins, forms, effects and normative stakes of this governing mode, and how can it be theorized in the global setting?

Though the topic has not had the attention it deserves, two recent books—*Emergency Powers of International Organizations: Between Normalization and Containment* by Kreuder-Sonnen (2019), and *Politics of Last Resort: Governing by Emergency in the European Union* by White (2019)—offer systematic studies of exceptional politics at a transnational level. As both authors argue, not only does the lens of emergency politics help clarify the governing of some recent crises, but it helps explain how international institutions and orders emerge over time. Emergency regimes tend to be normalized, and much of today's global institutional architecture is the legacy of yesterday's such measures. Emergency rule is a key way in which powers are redistributed in global politics—from national to transnational arenas, from legislatives to executives—as well as the basis of efforts to legitimize these transfers.

At its core, emergency politics is about a script of rule in which actions departing from convention are rationalized as necessary responses to exceptional and urgent threats. It denotes a set of *practices* on the one hand, in which actors break with legal rules or established norms in a supposedly temporary fashion, and a way of *rationalizing* such moves on the other hand as responses to extreme circumstances. Crises of all sorts can be the occasion for such moves—from famines to earthquakes, currency runs to pandemics—as well as pseudo-crises in which authorities exploit or construct an emergency situation. Among the rules and norms typically transgressed are the rights of individuals—of assembly, free speech, and so on—but also systemic aspects such as the separation of powers, the procedures of democracy, and the sovereignty of states.

In some ways, emergency politics has been central to international relations (IR) from the beginning. The archetypical emergency is war, and the modern state-system was built on the back of it. State institutions, bureaucracies, and other sites of authority emerged out of efforts to centralize power—and the resistance this generated—in the context of war preparation (Tilly 1992). Sovereign was the monarch who could raise an army, bring his nobility to heel, and defend his territory's borders in moments of extreme peril. Republics and city-states reluctant to go down this path found themselves absorbed or eclipsed over time (Spruyt 1994). War made emergency government, and emergency government made war.

In more recent years however, emergency politics has taken on new forms. While 9/11 and the “war on terror” have kept security issues to the fore, emergency politics has increasingly found application in nonmilitary settings, and has extended to supranational and intergovernmental institutions where coercive power is weaker or less visible. As Kreuder-Sonnen and White both argue, the transnationalization of emergency politics requires a rethinking of the concept and the dynamics surrounding it. One needs to decouple emergency rule from Schmitt's image of the sovereign dictator, as a dominant leader guiding the state through a period of danger, and engage with more diffuse forms of exceptional authority exercised at an inter-, supra-, or transnational level. Important also is to explore suspensions and transgressions not just of formal laws but uncodified norms, and to look beyond the context of war to the governing of the regional and global economy. For White, this means looking at how emergency powers have been produced and entrenched in the EU, feeding off a wider transformation of executive authority and with implications for populism and other aspects of democracy. For Kreuder-Sonnen, it means studying emergency empowerments of international organizations (IOs) such as the WHO and the UN Security Council comparatively, and analyzing the drivers of their longer-term institutional and normative consequences.

This Forum aims to be an entry-point into the analysis of emergency politics after a period of especially intense globalization coinciding also with its increased contestation. It tackles some fundamental questions concerning the concepts of emergency and emergency politics, debates the appeal of different accounts, and explores their potential application beyond the state. It examines the historical context of contemporary exceptionalism, and what the latter implies for the authority and legitimacy of European and global institutions. Further, it offers contending perspectives on the constitutional and political ways in which transnational emergency politics might be circumscribed. Lastly, on the assumption that in some form it is here to stay, the Forum examines what features demand further study. We believe transnational emergency politics has important implications for IR today, most obviously in the wake of the current pandemic, and our aim is to map out the key lines of the debate.

Our discussion has something of a Western bias. Emergency politics beyond the state inevitably tracks the emergence of political authority in international institutions, since it is both one of the mechanisms by which such authority is created, and one of the possibilities increasingly available thereafter. Hence, it is the most integrated organizations possessing supranational (delegated) and intergovernmental (pooled) authority, such as the EU, UN, WHO, and International Monetary Fund (IMF), which are likely to reveal the dynamics and legacies of transnational exceptionalism with particular clarity (see also Kreuder-Sonnen and White 2021). There is every prospect, however, that the template we describe may find future expression in other contexts of regional integration, from Mercosur and ASEAN to the African Union, entwined with distinctive features including those born of postcolonial ties. Further, the emergency empowerment of global institutions has implications far beyond the West, as suggested by the experiences of the IMF in South America during the economic crisis of the early 2000s (Klein 2008).

The Forum begins with a contribution by Séville, who provides a critical introduction to some of the main concepts related to exceptional politics. Highlighting those of crisis, emergency, and state of exception, she reviews their use (and abuse) in the classical context of the state. Steffek then discusses the conceptual transfer of the emergency problematique to the level of international institutions. Contrasting Schmitt's statist view to Kreuder-Sonnen and White's focus on international authority, he draws out the conceptual implications of redeploying the emergency framework in the transnational realm. Next is an intervention by Koenig-Archibugi, who lays out some of the normative stakes of exceptionalism beyond the state. Using the example of the EU, he examines transnational exceptionalism's effects on democracy, both in its *demos* and *kratos* dimensions. Heupel then moves the discussion to the analytical question of how to account for the normalization of emergency powers. Based on observations of the UN Security Council, she highlights the importance of three organizational practices that help supposedly exceptional arrangements to be perpetuated. That the normalization of emergency powers poses normative problems is Patberg's point of departure: he discusses potential avenues to constrain postnational exceptionalism legally and politically. The Forum concludes with a rejoinder by Kreuder-Sonnen and White, who respond to some of the issues raised and lay out avenues for future research on emergency politics after globalization.

## Why Emergency? Reflections on the Practice and Rhetoric of Exceptionalism



Astrid Séville

Ludwig-Maximilians-University Munich

For some scholars, the catastrophic earthquake and subsequent tsunami that consumed Lisbon in 1755 gave birth to modernity. Before such an incomprehensible and devastating disaster, philosophers like Kant, Voltaire, and Leibniz started questioning divine providence, morality, reason, and rationality. The event disclosed the absurdity and brutality of a world ripe for disenchantment, producing an awareness of profound insecurity—everything could change or tumble down. A narration of crises and their overcoming would become characteristic of modernity thereafter (Koselleck 1988). Public authorities would be compelled to “manage” such crises as natural disasters, financial crashes, political upheavals or revolutions—and they would need to decide on procedures to govern such critical situations. The litmus test for any political order is how it enables and constrains authorities in the governing of extreme circumstances. Should governments be allowed to suspend laws? What kind of (constitutional) roadmap for crises and emergencies can be developed? And, with regard to the lucid analyses found in White's *Politics of Last Resort* and Kreuder-Sonnen's *Emergency Powers of International Organizations*, how is crisis governance reshaped in an age of transnational and international authority?

Proponents of states of emergency and strong executive power, such as Carl Schmitt or Ernst Jünger, have often had an affinity for war, as Lenin had one for revolution. Revolutions and wars impose an all-encompassing purpose, a vision of “oneness.” Whereas modern societies are typically composed of a variety of subsystems—political, economic, legal, medical, aesthetic, and so on, each with different logics and programs (Luhmann 1995)—extreme circumstances are apt to counter this differentiation and evoke a unified collective beyond it. To be sure, recent crises such as the Euro crisis or the so-called migration crisis in 2015 were not at all comparable to war. They did not suspend ordinary functions, conflicts, and differentiation within society. But the Covid-19 pandemic has indeed been discussed as a moment of a genuine emergency, in which medical concerns trump political, economic, and educational considerations, recalling the natural disasters and crises that have recurred across modernity (Turner 2021). Hence, one might ask whether these circumstances fulfill the criteria for a state of exception—do we finally have good reasons to speak of *executive exceptionalism* in a post-war system?

In the following, I discuss these questions and make a case for a conceptual distinction between crisis and state of exception. Rejecting the notorious Schmittianism found in today's public debates, I argue that the current practice and rhetoric of exceptionalism as found in political discourse are misleading. By highlighting the particular case of Germany's management of the Covid-19 pandemic, I show that national institutions and norms may remain robust and resilient when their legal framework is well designed. This finding has implications for the normative problems linked to *transnational* emergency politics as raised by White and Kreuder-Sonnen (see also Patberg, this Forum).

### Emergency and the State of Exception

The pandemic that broke in 2020 and the crises cited above were all experienced as profound. They seemed beyond the grip of elected politicians. They shook up patterns of social action, exposing structural and systemic failures and problems. In short, circumstances appeared as constraining forces beyond political control. In a financial crisis, gaining and maintaining financial stability is a primary goal. In a global pandemic, containing the virus is the *raison d’état*. Such crises have a transnational dimension; financial markets are international, migrants, and viruses cross borders. Whether one approves of regional and global solutions or insists on national and local ones, recent crises have challenged the nation states’ capacity to solve problems unilaterally, and brought competing authorities at different levels to the fore. The transnational management of recent crises has exposed “a dispersed emergency regime rather than a clearly authored state of exception” (White 2019, 34). White's diction builds on a distinction, and it is helpful to elucidate concepts such as *state of exception, emergency*, and *crisis*.

The canonical text on states of exception is still Carl Schmitt's *Political Theology I* (1922). Schmitt, a German jurist and political theorist (1888–1985), approached emergency politics as a quasi-theological, mythical moment of anomie, in which an authority emerges that heroically creates (new) laws and a new status quo. For Schmitt, no legal norm can handle an emergency, and the strict application of “normal” law in extraordinary times could even make matters worse (Schmitt 2005). Instead, law will often need to be suspended, in what is known as a *state of exception*. Schmitt insists that such a process is impossible to codify—that it is about powerful individuals seizing the moment. Constitutions can define who will decide in the case of exception, but they do not provide public authorities with procedures as such. Schmitt famously characterizes the modern state in terms of this monopoly of decision. “Sovereign is he who decides on the exception” (Schmitt 2005, 5).

A state of exception is defined then by the idea that the constitutional order normally in force does not apply. To resolve a crisis, public bodies with the capacity to act swiftly and effectively are empowered, and rights are suspended to facilitate their actions. That Schmitt discards constitutional options for handling a state of exception, based on formulating legal conditions that constrain the emergency competences of political agents, limits the appeal of his account today. Schmitt's work is arguably not especially helpful for understanding today's “emergency politics” in consolidated, liberal democracies and in the transnational arrangements they participate in. It takes some conceptual twisting to make use of Schmitt's argument (see also Steffek, this Forum). His concepts and analysis are stylistically brilliant but compromised by his ideological leanings—by a reactionary, existentialist, and Catholic antiliberalism. Nothing in the Western hemisphere in recent times resembles Schmitt's idea of a state of exception; nothing has been radically exceptional and unforeseeable—and nothing has suspended all existing legal norms and revealed a genuine political sovereign beyond legality.^1^ Thus, for all the public talk of states of exception, recent circumstances should better be called *crises*.

### A Constructivist Reading: Crisis as a Moment of Narration and Intervention

The notion of crisis allows for a more pragmatic and less dramatic reading of today's events and their political handling in the national as well as transnational sphere. Complementary to the notions of emergency and exception, a crisis can be understood as “a process,” as “a moment of decisive intervention, a moment of transformation” (Hay 1996, 254). A crisis implies more than a rupture or breakdown: it refers to a situation in which agents—state agents, public and transnational authorities—need to intervene and make decisions. Any intervention requires an identification of the crisis, of its roots, and of possible dynamics and solutions. Political agents need to present a narration and interpretation of a crisis: “[s]uch narratives must recruit the contradictions and failures of the system” (ibid.). Failures are constructed and represented in crises—and defining a crisis means to frame the range of feasible or suitable coping strategies. Even necessities and constraints are politically manufactured. Consequently, one can call for narrations that name the structural, systemic, and endogenous roots of recent crises. They need to be put into context, and to find strategies for their resolution is to analyze and identify fundamental systemic contradictions and failures. To exceptionalize moments of crisis is to depoliticize the underlying issues.

Furthermore, political discourses that frame crises as exceptional emergencies play into the hands of agents, groups, authorities, and organizations that long for a vigorous, yet more authoritarian response. They facilitate efforts to sidestep norms, routines, and (legal) procedures and to exercise pressure on agents involved in decision-making processes. If politicians or representatives repeatedly refer to the truly exceptional nature of pressing issues and invoke emergency measures, they rouse a desire for an heroic, quasi-miraculous political response. They raise *neo-Schmittian* fantasies and suggest that political agents may (temporarily) suspend the differentiation characteristic of modern society.^2^ This is a fantasy of far-reaching political agency, of the ability to steer societies unbound by constraints.

And yet by citing necessity, functional demands and international limitations, and by muddling through with weak crisis management, political agents swiftly disappoint this desire. Indeed, speaking of necessities and urgency fosters a postheroic discourse at odds with political agency. Politicians themselves contribute to the impression that they are powerless, forced by events, disempowered by international obligations, pressured by factors beyond their control. The rhetorical invocation of constraints, inevitability and time pressure in turn provides a target for those who contest a “politics of necessity” with a “politics of volition” (White 2019). This interplay between neo-Schmittian fantasies of political primacy and managerial approaches focused on mere problem-solving seems to be symptomatic of modern politics (Nassehi 2012, 42), and the effect is to aggravate political conflicts and mobilize populists.

### The Role of Law: Germany's Resilience

It has been argued that speaking of emergency too hastily may be counterproductive. Against this backdrop, we might contrast the public talk of emergency and exceptional times with a perspective on the role of law highlighting its potential robustness in times of crisis. The example of German crisis management of Covid-19 shows that invocations of a state of exception are not only potentially dangerous but also out of tune with the astonishing resilience of law. The handling of the pandemic in this national context, as viewed at the time of writing (April 2021), is notable for having remained within the boundaries of legality. True, some rights have been curtailed. German regional governments have introduced restrictions and regulations, some even enforced by ministerial decree. But these actions have retained a legal basis in the German Infektionsschutzgesetz (the pandemic act) and thus remained within the bounds of the Basic Law. Contra Schmitt's expectations, the constitution has shown itself well prepared to legally contain and control an emergency (Kaiser 2020).^3^

German public opinion toward the government's policies has fluctuated. Protests against mask and lockdown policies have recurred, with political authorities and media accused of overreaction (Nachtwey, Frei, and Schäfer 2020). Support has also been affected by the problems of the vaccination program.^4^ But the country's constitutional foundations have remained strong. Citizens continue to invoke their fundamental rights, and courts have had to decide whether the restrictions conform to German (constitutional) law, from time to time ruling against them. The principle of proportionality has generally been complied with (Kersten and Rixen 2020). Legal scholars rightly stress that legislators and courts need to find answers to questions of appropriateness and to balance interests, and in Germany they arguably have done so. Once more, this highlights the importance of strong, independent courts—and particularly, the legitimacy and authority of a constitutional court. This is just one of the ways Germany's domestic crisis management benefits from features absent at the transnational level (see Patberg, this Forum). If emergency politics can still be constrained in the domestic setting, it is arguably on account of some of the very things missing in the transnational realm. Yet Germany highlights the kind of model that can work. Admittedly on the national scale and in a particular case, it shows the advantages of the constitutional inclusion of emergency rules instead of neo-Schmittian re-enactments of exceptionalism (cf. Ferejohn and Pasquino 2004; Gross and Ní Aoláin 2006).

Germany also exemplifies crisis management in a federal state where power is dispersed, and where (state) governments of different party affiliations need to cooperate while competing political motivations and designs collide. Whether the intricacies and benefits of federalism combined with a robust constitution can provide us with a model for multilevel, *transnational* emergency politics is a matter that needs further scrutiny (Kreuder-Sonnen 2019).

### Analyzing and Routinizing Crises

The current pandemic has shown that restrictions on freedom are possible under the rule of law. This suggests the conclusion that we need to discuss the possibilities of legitimate crisis management and how extraordinary means of crisis management can be legalized and justified. There are several ways in which constitutions and legal frameworks can domesticate emergency politics (Kaiser 2020). The legal accommodation of exceptional powers *may* prevent public authorities turning to the gray zones of the law or even to illegality. But, of course, the containment of emergency powers remains contingent on political practices, on the functioning of institutions, on the set-up of *international* institutions, and—yes—on the behavior of political agents within these institutions (Schindler 2014; Kreuder-Sonnen 2019).

Finally, another political way to contest exceptionalism and the politics of emergency is to insist on the (regrettable) regularity, recurrence, comparability, and recidivism of crises. As we have observed, modernity, democracy, and capitalism are crisis-ridden—what was true at the time of the Lisbon earthquake becomes only more so in the age of financial capitalism and the Anthropocene. This crisis-proneness is a structural characteristic of modern societies, and we should not fall into alarmism as soon as a new crisis emerges. Instead of inciting neo-decisionist fantasies by flirting with exceptionalism and talk of emergency, political discourse should tackle crises as ordinary yet critical junctures. One could formulate the paradox that crises must become a political routine to debunk the myths and temptations of exceptionalism.

The current pandemic has exposed the failings of contemporary societies. It has not, so far, resulted in authorities and agents demolishing liberal and democratic institutions (Ginsburg and Versteeg 2020). Nevertheless, our analyses need to expose power shifts between institutions, organizations, and branches in the political system, as well as the dispersion of power in informal, opaque, and unaccountable forums. Contesting such power shifts in national as well as transnational governance architectures would be aided if public discourse did not perpetuate the hasty narratives of emergency and exceptionalism that support them.

## The Liberal Taming of Carl Schmitt: Emergency as Rhetoric and Policy Style



Jens Steffek

Technical University of Darmstadt

In this contribution, I explore the radical re-conceptualization of “emergency” that we find in contemporary treatments of emergency politics. It is almost a century now since Carl Schmitt prominently introduced the concept of emergency to constitutional analysis. In Schmitt's work, the emergency is a volatile situation in which the survival of the state is at stake and crushing the opposition the last resort of sovereign power. This contrasts sharply with the idea of emergency politics that we find in recent books by Kreuder-Sonnen (2019) and White (2019). Both authors use the notion of emergency politics in what I would call a Lockean discourse of grievances, denouncing the encroachment of transnational executive powers on human rights and parliamentary democracy. Emergency politics is conceived here as a particular policy style, but also a rhetorical strategy of the executive that seeks to push through controversial measures. That conceptual shift is accompanied by a redefinition of power that takes us from the “power to coerce,” as it was in Schmitt, to the “power to persuade” (Oppenheim 1978, 590). In what follows, I will first outline further Schmitt's notion of emergency, then scrutinize its re-conceptualization by Kreuder-Sonnen and White. In the last section, I suggest that despite some fundamental differences there also is common ground between the two positions.

### Emergency in Schmitt

In the first chapter of his *Political Theology*, Schmitt famously sentenced that “sovereign is he who decides on the exception” (Schmitt 2005, 5). That “he” for Schmitt was indeed an individual, a person taking decisions on behalf of a state. Emergency situations have an air of existential contingency and danger about them. In the emergency, the very survival of the state is at stake, and suspending the normal constitutional order is justified as an act of *raison d’état*. An emergency reveals the real seat of power, because it puts to a test if “he who decides” is able to coerce his opponents. The power in question is Hobbesian *auctoritas*, the ability to command, to coerce and if necessary to kill. Even if he was a jurist, Schmitt in fact defended a crude legal realism, in which all law, at the end of the day, is a function of executive power. As he infamously put it in 1934 while the Nazis established their brutal dictatorship in Germany: *Der Führer schützt das Recht* [The Fuhrer protects the law] (Schmitt 1934).

Schmitt's analytical perspective on the emergency situation also needs to be seen in the context of his antiliberal partisanship. Schmitt was an eminently political thinker who deployed his theoretical arguments strategically and polemically (Teschke 2011). Schmitt's intellectual opponent was a Lockean liberalism that wanted, by means of a legal order, to impose limits on state power and safeguard citizen rights. This Anglo-American *normativism*, as he usually called it, was Schmitt's central political target (Scheuerman 1996). Schmitt's critique of liberal law had an international dimension too (Koskenniemi 2016). The collection of essays from the 1920s and 1930s, where his views on IR unfold, alludes to Schmitt's “fight against Weimar, Geneva, and Versailles” already in the title (Schmitt 1994). International treaty regimes and organizations for Schmitt were manifestations of Anglo-Saxon liberal normativism at the international level.

Accordingly, Schmitt's goal was to undermine the legitimacy of the Treaty of Versailles (denounced as a victors’ “Diktat”), the League of Nations and the Kellogg-Briand Pact. To be sure, among German international lawyers of the Weimar republic that intellectual purpose was nothing unusual (Steffek and Heinze 2020). Schmitt's ideas were taken up by revisionist international lawyers who argued against international organization, collective security and disarmament treaties. Their line of argument was straightforward and largely analogous to Schmitt's. International law was a product of the power constellations of a certain historical situation. The victors in war shaped the international order, and multilateral IOs such as the League were their handmaidens. Revisionist German scholarship used this line of argument to justify breaches of international law and Nazi military aggression (Berber 1939; Bilfinger 1940). Disregarding an unjust and illegitimate international legal order whenever power constellations allowed for it was justified, even imperative, from the perspective of *raison d’état*. Legal realism and political revisionism here went hand in hand (Jütersonke 2010).

### Redefining Emergency and Sovereignty

Such, historically, were the uses of Schmitt's international thought in an attack on the “Wilsonian” conception of international organization and law in general, and on the League in particular. It is intriguing to see, therefore, how recent approaches in the field of IR and European studies have borrowed Schmitt's emergency semantics when analyzing IOs and the EU. First, *analytically*, Schmitt probably would have denied that IOs have anything like real power over their member states. His musings on the emergency situation focused on the state where formal sovereignty and the power to coerce continue to reside. Kreuder-Sonnen and White, in contrast, argue that IOs and the EU have quite some power over states and citizens. Second, *politically*, Schmitt and friends were antiliberals and anti-internationalists. In contradistinction, Kreuder-Sonnen and White seem to support the ideals of international cooperation and an international rule of law. However, they both have their misgivings. They use the semantics of emergency and state of exception to denounce attacks on individual rights by IOs (Kreuder-Sonnen) and on parliamentary democracy (White). For Schmitt, arguably none of this would have been a concern.

To use Schmitt's conceptual legacy against his own intentions, some conceptual moves are necessary. First, both authors find it necessary to deconstruct Schmitt's sovereignty-emergency nexus, as IOs quite clearly do not have Hobbesian *auctoritas*. Their authority resides in a far more dispersed and amorphous multi-executive, or “multi-bureaucratic” form of governance (Held 1991, 146). Kreuder-Sonnen characterizes it as follows: “In the context of global governance [...] hierarchical relations exist primarily in segmented *spheres of authority* that rely on recognition and acquiescence by the authority-addressees without (necessarily) providing for means of enforcement” (Kreuder-Sonnen 2019, 38). Segmented authority without means of enforcement against resistance is, in a way, the conceptual counterpoint of Hobbesian *auctoritas*. “IO exceptionalism,” then, “rests on the complex formal and informal, direct and indirect power relations of composite actors” (Kreuder-Sonnen 2019, 40). In a similar vein, White speaks of “emergency politics informally co-produced by the many” (White 2019, 3).

The second key move is to redefine emergency. From Schmitt's objectively given situation of extreme confrontation on the brink of civil war, emergency morphs into a specific rhetorical style supporting a certain kind of governing practice. Executives use the emergency as an argument to justify their policy choices, or to seize new competences. If successful, such moves offer windows of opportunity for achieving rapid change and/or rescuing the status quo, depending on the political intentions of the executive. White writes of that process: “Emergency rule involves appeal to exceptional circumstances as the basis for exceptional measures. It involves identifying a sudden onset of problems, leading to situations out of keeping with the world as it was before. Present and past are cast as discontinuous, and the unexpected character of the challenges arising is emphasized” (White 2019, 109). Executives thus seek to turn a moment of crisis into a TINA situation, where there is no plausible alternative to what the rulers are suggesting.

At the hands of constructivists, Schmitt's existential struggle over *auctoritas* becomes what Kreuder-Sonnen calls a “rhetorical power game” (Kreuder-Sonnen 2019, 59), in which one or more sides try to win the public's consent for their emergency measures. No situation is objectively an emergency, it all depends on the rhetorical framing and what values are prized over others. The constructivist account of emergency thus cleanses that concept of traditional connotations with violent struggle, crushing resistance, and subjugating enemies. The power in question here is not the power to coerce but the power to persuade and deceive. The task of critical political science is to expose these techniques of the executive. This brings me to the last point, which is a subcutaneous sympathy for Schmitt's ideas, at least in White's book.

### Common Ground: The Critique of a “Science of Politics”

I think it is appropriate to say that Kreuder-Sonnen's book is written from a liberal-internationalist perspective, committed to multilateralism and the rule of law in international affairs. That still allows for some criticism of IOs. Kreuder-Sonnen argues in his conclusions that IO emergency powers can in principle be justified because they may, for instance, break up gridlock situations. The normative requirement is that these interventions be proportional to their goals (Kreuder-Sonnen 2019, 200–1). White seems to be more critical of the EU and its exceptional politics. He wants to save parliamentary democracy from being hollowed out by the forces of executive internationalization. His support for international organization is more guarded than Kreuder-Sonnen's, and seems conditional on how democratically international governance unfolds.

White's criticism echoes an aspect of Schmitt's critique of liberalism that McCormick (1997) has emphasized: a critique of managerialism and a conception of politics as science and technique. As a student of Max Weber, Schmitt reflected on the consequences of rationalization and came to oppose the relentless advance of bureaucracy into the political realm. White equally rejects predominantly technocratic, “scientific” styles of policy-making (White 2019, 125) and here seems to be in agreement with Schmitt. To make his case, however, he marries politics and technocracy. This is unconventional and deviates from Weber's and Schmitt's views. As is well known, Weber made quite a stark distinction between the spheres of politics and bureaucracy as “diametrically opposed pure types” (Mommsen 1989, 46). On the one hand, there is the political realm of power struggle but also of creativity, innovation and change; on the other, the “continuous rule-bound conduct of official business” (Weber 1978). Elsewhere, I too have defended the view that public IOs are a force of de-politicization. I read them as extensions of Weber's *Fachbürokratie* (technocracy) to the international level (Steffek 2017). Interestingly, White abandons that conventional mode of pitching volitional politics against rule-bound administration. Neither does he buy into the idea that in emergency situations, when quick and resolute action is needed, politicians gain the upper hand and marginalize the technocrats.

Rather, he argues that politicians and IO technocrats are complicit in manufacturing or at least exploiting emergency situations. “It becomes clear there is no paradox in the fact that the same technocrats who feel able to break with the existing order in exceptional times may have had a hand in its prior construction” (White 2019, 116). Together, politicians and technocrats use the window of opportunity that emergency offers to deeper entrench a largely unchanged political program. Technocrats can, following White, exploit the emergency situation by offering their know-how (White 2019, 119). That way, the EU technocracy attains a role as emergency manager alongside national executives, and becomes complicit in, rather than victim of, the emergency momentum. White thus recasts the scene and can position this executive alliance against “the people,” reproducing a well-known populist figure of thought: the revolt of the sovereign people against the usurpation of power by a bureaucratic elite. That recovery of political sovereignty from usurpation is what unites, on an abstract level, Schmitt and White. Of course, Schmitt wanted the authoritarian leader to perform the sovereign's will (Wolin 1992, 425), whereas White wants to revitalize democracy.

To conclude, I have traced in this contribution a conspicuous shift in the definition of emergency. Schmitt defined the emergency as a moment of existential struggle that revealed the real distribution of power in a polity. That struggle was objectively given, not a matter of perception or narrative framing. Schmitt strategically deployed his concept of the emergency situation in an attack on liberalism, parliamentary democracy and the rule of law. For Kreuder-Sonnen and White, in contrast, an emergency is not objectively given but socially constructed, and in part a political device that the governing elite uses to minimize resistance. They analyze emergency politics to expose these tricks of the executive and to warn, in a broadly liberal tradition, against threats to individual rights and parliamentary democracy. The shift in perspective is thus quite radical. While Schmitt conceived the emergency situation to defend transgressions of the executive, current scholarship uses the term to denounce them.

## Democratic Deficits in the Transnational Politics of Emergency



Mathias Koenig-Archibugi

The London School of Economics and Political Science

Most readers of this Forum might agree that transnational emergency politics is bad for democracy. But *why* is it? This contribution compares two broad answers to this question. Each focuses on a different basic ingredient of democracy (*δημοκρατία*): *δῆμος* (the commons, the people) or *κράτος* (power, rule). If democracy means rule of the people, the democratic quality of a political system or process involves two distinct judgments. The first is about whether the people *rules*. We can call this the *kratos* question. The second judgment is whether rule is exercised by an appropriately defined *people*. We can call this the *demos* question. The first broad answer to why transnational emergency politics produces or exacerbates a democratic deficit focuses on the *kratos* question: it is bad because it limits the capacity of the people to rule. By contrast, the second answer focuses on the *demos* question: it is bad because it draws or reinforces boundaries around the *demos* or *demoi* in a way that cannot be justified.

Kreuder-Sonnen's *Emergency Powers of International Organizations* (Kreuder-Sonnen 2019) and White's *Politics of Last Resort* (White 2019) provide insightful analyses of transnational emergency politics that emphasize the *kratos* question. Both books highlight the Euro crisis of the 2010s as a prominent manifestation of emergency politics and a challenge to democracy in Europe. While Kreuder-Sonnen concentrates on the dynamics of self-empowerment by IOs, White examines how an array of executive actors in Europe colluded in enacting and justifying emergency rule. This contribution will refer primarily to White's argument. His account shows how the mechanisms of securitization and disavowal of agency played a prominent role in how the Euro crisis was managed. White argues that “executive institutions seek in the politics of emergency an authority of last resort, one that can address some of the concerns of public opinion while responding to demands that are generated *elsewhere*” (White 2019, 66, emphasis added). This “elsewhere” is, ultimately, the realm of private investors, who stood to benefit most from the priority accorded to financial stability by the authorities in charge of emergency rule (White 2019, 23). In White's account, governing by emergency undermines the democratic process by insulating key decisions from the domain of public deliberation and accountability, and it hinders democratic outcomes by privileging the protection of narrow interests over those of the public.

Executives are not forced to behave in this way. White singles out the Greek executive led by the Syriza party as an instance of a government willing to challenge the system of emergency rule. At the height of the bitter negotiations over a third bailout for Greece in the summer of 2015, Syriza called a referendum on whether Greece should accept the conditions requested by the “Troika” [European Central Bank (ECB), European Commission, and IMF] on behalf of the other members of the Eurozone. White notes: “although Syriza ultimately did little to build on the vote, it was a form of authorization nonetheless. Executive discretion in the Euro crisis, by contrast, was generally the action of elites acting independently of public control mechanisms, and to the extent that they had an electoral mandate at all it was one weakly linked to the measures advanced” (White 2019, 159). In this interpretation, the approach chosen by the Syriza executive was atypical: by wielding the power of exception, manipulating perceptions of what was possible and colluding with each other, most European governments weakened democracy because they undermined the ability of the people to *rule*.

How persuasive is this answer? The Eurozone had nineteen members in 2015, and it is not possible here to survey to what extent each of them acted independently of public control mechanisms and detached from an electoral mandate. I will focus on the case of Germany, because it is widely regarded as the main antagonist of Greece in the Euro crisis and because the drivers of its policy have been studied extensively. It is worth noting that Germany's key players pointed at domestic mandates and constraints not only in public but also when negotiating behind closed doors. When Syriza's finance minister told his German counterpart that the newly elected Greek government had a democratic mandate to oppose loan conditions requiring further “austerity,” the latter retorted: “It is my mandate against yours” (Varoufakis 2017). Four years earlier, during tense negotiations at a G20 summit dealing with sovereign debt problems, the presidents of the United States and France had tried to press Germany's chancellor into agreeing to provide financial support for Italy without the guarantees that she demanded. She had reacted angrily, exclaiming “It's not fair, I'm not going to commit suicide!” (Spiegel 2014). The context made it clear that she meant political suicide at the polls.

Finance minister Schäuble and Chancellor Merkel were not trying to deceive their interlocutors. A Eurobarometer survey fielded a year before the Chancellor spoke those words, and a few months after the first bailout of Greece in 2010, shows that the German public was roughly evenly split on whether, “in times of crisis, it is desirable for [Germany] to give financial help to another EU Member State facing severe economic and financial difficulties” (46 percent agreed, 45 percent disagreed, the rest did not know). Moreover, when asked whether, “to emerge from the crisis rapidly, EU Member States should first reduce their public spending or should they first invest in measures to boost the economy,” German respondents favoring austerity outnumbered those favoring a fiscal stimulus (European Commission 2013). A survey fielded in early 2012, a few months after the second bailout for Greece, shows that the Germans who were against bailout payments for overindebted EU countries outnumbered those in favor (61–28 percent) (Bechtel et al. 2014). If, like Syriza, the German government had called a national referendum on the bailout packages negotiated by Eurozone members, rejection would have been very likely. What the German government was willing to agree to—bailout funds conditional on the Greek government cutting spending and on banks and other holders of Greek debt losing half of the value of their loans—seemed just within the bounds of acceptability to the German median voter, although still electorally risky (as the rise of the anti-Euro *Alternative für Deutschland* party would soon confirm). A detailed process-tracing study of German policy-marking in the Euro crisis concludes that “the German government, despite intensive lobbying efforts by banks and industry associations, responded rather closely to the demands of the public” (Degner and Leuffen 2019, 1), and that this close match was driven by electoral considerations.

Was the responsiveness of the German government exceptional, like Greece's? Determining the congruence of executive policies and the “popular will” is more difficult for other countries, but there are indications that it was fairly high. An analysis comparing 24 countries shows that the share of the population favoring a reduction in public spending, that is, austerity, when asked the Eurobarometer question already mentioned is a reliable predictor of their government's support for the hawkish positions held by the German government on various Euro crisis-related issues (Armingeon and Cranmer 2018). Low economic vulnerability to the risk of austerity (and consequently to electoral punishment) is another reliable predictor of hawkish positions, whereas exposure of the country's banks to sovereign debt is not. In other words, European governments seem to have approached the Euro crisis holding positions that they felt able to justify to voters at the next election.

These government positions also circumscribed what supranational actors were able to do. It can be argued that the role of the Commission was mostly limited to implementing (and offering some ex post justification to) the policies that emerged from intergovernmental negotiations (Blustein 2016). The ECB was more proactive in creating the so-called Outright Monetary Transactions (OMT) program that enabled it to purchase bonds issued by Eurozone governments in distress. As Kreuder-Sonnen (2019, 141–3) shows, however, both debtor and creditor governments supported this program, whereas parliamentarians who opposed it represented a small share of the electorate in Eurozone countries. It may well be that “the creditors’ rationale was to reduce political responsibility and domestic contestation by passing some of the burden on to the *apolitical* ECB” (Kreuder-Sonnen 2019, 141), but even that would not necessarily stop us from concluding that electoral accountability was an essential driver of decision-making during the crisis.

Assuming this is all true, does it mean that there was no democratic deficit in the politics of the Euro crisis? Not necessarily. This contribution started by pointing at two approaches to why transnational emergency politics is bad for democracy. The second approach might concede that governments were by and large responsive to their electorates, but it stresses that it was mainly or exclusively *to their nationally defined electorates*. In the second interpretation, emergency politics does not generate a democratic deficit (primarily) because it disables the mechanisms by which the people can exercise their right to rule, but because it promotes a type of politics that privileges nationalist and exclusionary understandings of the *demos*.

Scholars of IR may object that it would be naïve to expect otherwise. As Martin Wight (1978, 95) put it several decades ago, “a Foreign Minister is chosen and paid to look after the interests of his [*sic*] country, and not to be a delegate for the human race.” But this objection is not compelling, at least in the case of the EU. By the time the Euro crisis erupted, nearly half a century of institutional developments had already eroded the idea that EU politics was simply a matter of governments bargaining on behalf of domestic interests. To be sure, that always remained an important part of the story, and the institutional setup of the EU reflected this reality by giving pride of place to the European Council and the Council of Ministers. But other elements of the European governance architecture could not be reduced to intergovernmentalism: the expansion of weighted majority voting, the growing power of the Parliament, the tendency of its individual members to vote along partisan lines more often than national lines, and the expectation that supranational institutions such as the Commission and the Court of Justice should pursue some kind of pan-European goals. This does certainly not mean that the institutions of the EU expressed a homogeneous European common interest, any more than national governments can be said to express a homogeneous national interest. It means that European governance was quite advanced in its ability to organize and structure political conflicts stemming from multiple overlapping cleavages, instead of being fixated on cleavages defined by national borders. Given that the values and interests of Europeans are empirically distributed in a way that criss-crosses national borders instead of tracking them (Hale and Koenig-Archibugi 2016), the complex institutional system of the EU based on the so-called “Community method” promised a more faithful aggregation of the diverse political preferences of Europeans than a purely intergovernmental system. The Community method both presumes and constitutes a complex European *demos* that matches the actual structure of power, interdependence, interests and values on the continent.

From a *demos* perspective, transnational emergency politics is bad for democracy because it generates a vicious circle. On the one hand, the perception of an emergency promotes a reduction of complexity, puts executives in the spotlight compared to other branches, and (given a very weak executive at the transnational level) it elevates the national dimension over all others. When not resorting to unilateralism, key decisions are taken in summits where executive leaders are expected to defend the “national interest” against the claims of “foreigners.” Given the atmosphere of crisis, politicians who point at the interests and perspectives of those foreigners risk looking disloyal and treacherous. To be safe, even arguments for cooperation and accommodation are then framed with reference to the interests of the national *demos*, in the same way as arguments for confrontation are. On the other hand, intergovernmentalism as a decision-making mode exacerbates the sense of emergency itself. Compared to voting procedures distributed among multiple chambers, bargaining as a decision-making mode encourages players to engage in brinkmanship—key decisions are left open until the last minute, since both sides need to demonstrate resolve to domestic and international audiences. If the Euro debt negotiations at times felt like the Cuban Missile Crisis, it is because intergovernmentalism tends to transform every policy issue into a game of chicken.

To join up the *demos* and the *kratos* questions outlined at the beginning, we could ask how events might have unfolded if political decision-makers had combined responsiveness to the popular will with the principle that the relevant *demos* had to be Europe-wide. Elsewhere (Koenig-Archibugi 2019), I use Eurobarometer questions to conduct a thought experiment. If key policy-makers had been operating under a mandate to address the crisis in a way that reflected the “will” of the largest number of EU citizens regardless of how that number was distributed geographically across member states, how would they have handled the Euro crisis? In this exercise, a policy package that combined financial assistance for the worst-affected countries with a fiscal stimulus would have commanded a relative but not an absolute majority. Each of the other possible packages (a bailout conditional on austerity policies; austerity without a bailout; a stimulus without a bailout) was supported by a substantial proportion of European citizens. We know the policies that prevailed in the clash of national executives eyeing national elections, following the “my mandate against yours” principle, and wielding unequal economic power. But we do not know for sure what outcome would have emerged from a genuinely pan-European democratic process—which would have been precisely the beauty of it.

## On the Normalization of Emergency Powers: Observations from the United Nations Security Council



Monika Heupel

Otto Friedrich University Bamberg

Another normative problem of emergency politics beyond the state is the tendency of exceptional measures and powers to be normalized over time (Kreuder-Sonnen 2019). The United Nations Security Council (UNSC) provides a pertinent example. In its fight against terrorism from 2001 onwards, it assumed the power to decree legislative emergency measures for the international community as a whole. That is, the UNSC enacted resolutions that prescribe changes in UN member states’ domestic legislation. After the first adoption of a legislative resolution in the context of 9/11, the Council successfully repeated the endeavor in 2004 by passing resolution 1540 (UNSCR 1540) that enjoined sweeping obligations on all member states to inhibit the proliferation of weapons of mass destruction to nonstate actors. As it meant moving beyond a singular exception and recognizing the Council's power to enact global emergency law, UNSCR 1540 was initially very contested.

Shortly after the UNSC began to enact legislative resolutions, a debate emerged among International Law scholars about whether the UNSC had overstepped its legal mandate. Those who believed it did argued that the UNSC was only authorized to spring into action in conjunction with specific threats and, as a consequence, can only impose specific rather than generic obligations (Happold 2003; Elberling 2005). Alvarez (2003) even referred to the Council's legislative resolutions as a form of hegemonic international law that threatened to undermine the Council's legitimacy. Remarkably, though, most UN member states, after initial criticism, soon ceased to challenge the UNSC's authority to enact UNSCR 1540 (e.g., UN Security Council 2014a). Hence, UN member states for the most part did not keep up their objection to the UNSC assuming emergency powers. They accepted that the UNSC adopted exceptional measures justified by the existence of an emergency situation, namely the risk of weapons of mass destructing falling into the hands of terrorists.

Why did UN member states accept the UNSC assuming “legislative emergency powers” (Kreuder-Sonnen 2019, 87) by adopting UNSCR 1540? For Kreuder-Sonnen the answer is factual normalization as the result of a ““rhetorical power game” in which the actors try to convince the relevant audience of the appropriateness of their ... position” (ibid., 51). Accordingly, UN member states accepted the UNSC's assumption of emergency powers because the “pro-ratchet coalition,” that is the states that strongly supported the resolution, “could credibly deploy arguments of necessity in the face of an enduring crisis” and could portray the measures as proportionate given the gravity of the crisis (ibid., 82). The pro-rollback coalition, by contrast, that is the states that opposed the resolution, presented arguments relating to the excessiveness of the costs the intrusive measures created and the measures’ dysfunctionality. Yet, they could not find sufficient support for their arguments (ibid., 92–5) and, moreover, had to argue from an inferior power position, as the pro-ratchet coalition comprised the five veto-holding permanent Council members (P5) (ibid., 89).

Kreuder-Sonnen rightly draws our attention to factual normalization as a mechanism that can explain the entrenchment of emergency powers, and his discourse-based proportionality theory of IO emergency powers is highly illuminating. Yet, by putting the “rhetorical power game” center-stage, he inevitably has to neglect the nondiscursive practices that have also helped the UNSC to normalize its adoption of UNSCR 1540 and other legislative resolutions. In this contribution, I zoom in on three such nondiscursive normalization practices, namely association, emulation, and cooptation. I argue that these practices have helped the Council quell potential opposition and enlist at least limited support for its advance into legislation. They have, alongside Kreuder-Sonnen's rhetorical strategies, helped the Council to present its new powers as normal authority and consolidate the “new order” by “build(ing) public confidence” in it (White 2019, 88).

### Normalization Practices

#### Association

Political actors frequently try to gain legitimacy by associating themselves with presumptively prestigious actors (Hurd 2002; Bäckstrand and Kylsäter 2014). For instance, nongovernmental organizations (NGOs) seek to gain access to IOs so as to be perceived as trustworthy actors. Conversely, IOs interact with NGOs for the same purpose, when they give access rights to NGOs with the expectation that this will increase their own perceived legitimacy (Zürn 2012). Beyond legitimation, partnering with actors who are already established in a field can also make the actions of a newcomer appear normal. Thus, IOs that wish to make their assumption of emergency powers appear normal can associate themselves with actors that have already established themselves in the field into which they have advanced. By securing the endorsement of established actors they try to normalize their use of extraordinary measures. The approach to implementation chosen by the 1540 Committee—the Committee composed of all UNSC member states set up to oversee implementation—heavily leans on association. The Committee website contains numerous references and links to focal institutions of the nonproliferation regime (UN 1540 Committee 2020c). Moreover, to help states implement the resolution's far-reaching obligations, the 1540 Committee acts as a clearing house that brings states in need of assistance in contact with IOs and NGOs that can provide such assistance (UN 1540 Committee 2020a). Obviously, the main purpose behind this arrangement is to ensure states in need of assistance get it. Yet, it also enables the UNSC to associate itself with the focal actors in the field—and thus normalize UNSCR 1540.

#### Emulation

To portray themselves and their behavior as normal, political actors also emulate the behavior of relevant others. As scholarship on isomorphism has shown, organizations tend to model their procedures and practices on templates they find in their environment, especially in prestigious organizations (DiMaggio and Powell 1983), frequently motivated by a desire to increase the organization's legitimacy (Meyer and Rowan 1977; Suchman 1995, 589). At the same time, copying procedures and practices from other organizations that apply them routinely may also help IOs that have assumed new competences make their conduct appear normal. As such, following scripts from other IOs may help them signal to their member states and other relevant audiences that their actions are in the realm of normal governance and not extraordinary, even though they might be novel for them. Emulation is a central feature of the UNSC's normalization strategy, as the 1540 Committee draws on managerial implementation strategies UN member states are familiar with from their routine interactions with other IOs. For instance, states have to submit reports that document their implementation efforts (UN 1540 Committee 2020d)—an obligation states are familiar with from their interactions with the UN human rights treaty bodies and numerous other international bodies. Moreover, the 1540 Committee has gathered a set of best practices and has compiled so-called Matrices for each member state that give information on its implementation efforts with regard to different indicators (UN 1540 Committee 2020b). Thus, the 1540 Committee avails itself of “governing through goals” and has therefore adopted an approach states know from the implementation of the UN Sustainable Development Goals and various other contexts (Kanie and Biermann 2017).

#### Cooptation

Looking normal also implies that potential opposition is muted, for if there is constant opposition to an institution or its policies it is difficult to pretend that the way the institution acts or the policies it applies are ordinary. Thus, potential opponents need to be appeased to maintain an aura of normalcy. Cooptation is one important means to stifle or prevent opposition. It rests on a bargain between a power holder, the cooptor, and a (potential) challenger, the cooptee. The cooptor grants the cooptee privileges; in return, the cooptee provides support and displays loyalty (Gerschewski 2013; Kruck and Zangl 2019). The UNSC has relied on cooptation to foster support for UNSCR 1540. As regards states, the 1540 Committee has provided incentives for governments that had initially been sceptical of the approach taken not to challenge UNSCR 1540. Even though the UNSC has the power to adopt sanctions against states that refuse to implement its resolutions, it has nevertheless refrained from punishing intentional noncompliance (Heupel 2008).^5^ As a consequence, even noncomplying states can portray themselves as good citizens that honor their international obligations. IOs, NGOs, and academics, some of which had initially disapproved of the Council's encroachment on other actors’ competences, were also given incentives to support the Council's approach. IOs and NGOs that had long hoped for an opportunity to assist states in beefing up their nonproliferation capabilities were provided with access to states that suddenly had a legal obligation to strengthen their domestic proliferation safeguards. Academics were invited to join a Group of Experts and to participate in recurrent reviews of the status of the implementation of the resolution, which, again, provided them with access they did not have before.

#### Conclusion

I have shown that the UNSC uses three nondiscursive practices—association, emulation, and cooptation—to make its move into legislation with UNSCR 1540 appear normal and prevent contestation. In doing so, I have complemented Kreuder-Sonnen's proportionality theory of IO emergency powers, according to which IOs normalize their use of such powers by providing compelling arguments regarding the necessity, functionality and/or level of intrusiveness of their exceptional measures. Complementation is not to mean, however, that discursive and nondiscursive normalization practices operate in isolation from each other. Rather, the nondiscursive practices I describe provide the context in which Kreuder-Sonnen's “rhetorical legitimation contests” (Kreuder-Sonnen 2019, 51) are enacted. Association and emulation serve, at least in part, to lull UN member states and make them perceive the Council's emergency measures as normal—and hence to dissipate incentives to rhetorically challenge them. Furthermore, not punishing member states for intentional noncompliance serves to prevent states from worrying about the intrusiveness of the exceptional measures taken, and thus to coopt them in a wider sense, and, at the same time, deprives states of the argument that the Council's approach violates the proportionality principle. Hence, it is important to consider both types of factual normalization, discursive and nondiscursive, and how they relate to each other, to understand how IOs make the emergency powers they assume as exceptional measures seem normal.

For the case at hand, one has to be careful not to overestimate the scope of the normalization of the UNSC's emergency powers. The fact that states accept a specific application of an emergency measure does not imply that they generally accept the measure. Applied to the UNSC's legislative emergency powers, this means that states accepting UNSCR 1540 as normal does not mean that the general approach of adopting legislative resolutions is accepted as normal. In fact, the UNSC has only adopted two other legislative resolutions (including their follow-up resolutions), that is resolution 1373 on terrorism generally (UN Security Council 2001) and resolution 2178 on foreign terrorist fighters (UN Security Council 2014b). It therefore seems that states accept that the Council assumes emergency powers in limited circumstances, but not generally. This does not mean, though, that such a bounded normalization of emergency powers is not meaningful. On the contrary, having successfully normalized a specific instance of “decree legislation”, the UNSC has created for itself a reservoir of emergency powers. In light of the current tensions among the P5, this reservoir of emergency powers is at present no more than a reservoir in waiting. Yet, it may be activated in the future if the tensions subside or if issues arise in relation to which the P5’s interests nevertheless coincide.

## Toward a Democratic Response to Emergency Politics in the European Union



Markus Patberg

University of Hamburg

White's *Politics of Last Resort* and Kreuder-Sonnen's *Emergency Powers of International Organizations* decisively advance our understanding of a troubling phenomenon of contemporary politics: the self-empowerment of international institutions by means of emergency politics, which takes place in periods of crisis. In this contribution, I focus on the case of the EU, which both White and Kreuder-Sonnen analyze with an emphasis on the Euro crisis, and ask, from the perspective of political theory, what a democratic response to emergency politics might look like. Ultimately, all strategies discussed here face the problem that citizens lack opportunities to take the initiative in shaping the EU constitutional order.

Both White and Kreuder-Sonnen raise concerns about the transformative power and lasting effects of emergency politics with regard to the EU political system. As they explain, the Euro crisis enabled executive agents to bring about constitutional change without having to face the challenge of generating adequate democratic input legitimacy for it (Kreuder-Sonnen 2019, 117–23, 135–9; White 2019, 16–30; cf. Koenig-Archibugi, this Forum). Although new rules and institutions were initially introduced with reference to the “existential threats” of an exceptional situation, they quickly became firmly established. In other words, we witnessed a re-organization of public authority insufficiently (one might even say: not at all) grounded in democratic constituent power—a fact that did of course not prevent actors such as the ECB from claiming to be acting on behalf of “We, the People of Europe” (Lokdam 2020).

Now, if we follow White in assuming that EU emergency politics is a “persistent possibility,” because it is something to which the EU is structurally vulnerable, then a key question for those concerned about the EU's legitimacy is what democratic means there are to address such developments (White 2019, 50). In other words, the question is: if we have to assume that EU emergency politics will be a recurrent phenomenon, how can we ensure that citizens do not become passive recipients of crisis management? With reference to ideas outlined or prefigured in the books of White and Kreuder-Sonnen (see also Lazar 2009), let me consider three potential avenues for a democratic response to emergency politics: pre-emptive regulation, constructive re-appropriation, and targeted disintegration.

*Pre-emptive regulation*: A first option is to try to regulate emergency politics ex ante. As Kreuder-Sonnen indicates, this could take the form of an emergency constitution—presumably to be produced by means of a democratic procedure, a far from trivial precondition—which determines rules for the activation and exercise of emergency powers (Kreuder-Sonnen 2019, 208; Kreuder-Sonnen 2021). Such a mechanism could be enacted in the face of any transnational crisis for which the EU has relevant authority—those of the economic domain especially but not only. The intuitive appeal of this approach lies in the fact that a form of emergency politics based on democratic rules would not have to be regarded as executive self-empowerment. Rather, it could be understood as being constitutionally authorized. EU emergency politics could be given clear procedural and substantive boundaries whose observance could be subjected to judicial review.

While appealing, this idea has its limitations. One aspect to consider is who would be able to enforce an emergency constitution—or at least determine its violation in concrete cases. Presumably, this would have to be the task of the Court of Justice of the European Union (CJEU). The problem is that when courts are asked to decide on the legality of measures taken in an acute and ongoing crisis, they act under high external pressure. As the response to Covid-19 in many countries makes clear, states of emergency are not only situations in which the powers of the judiciary can be curtailed, but also moments in which courts often refuse to constrain the executive (Grogan 2020). The fear of hindering effective problem-solving can play a role here, as can the (perceived) lack of information and expertise needed for a targeted intervention. Considering the CJEU's role in the Euro crisis, especially its ruling on the OMT program, there is reason to fear that an EU emergency constitution could prove toothless. As Kreuder-Sonnen himself points out, the CJEU's reasoning in the OMT judgment “was not free from political constraint,” as the court practically deferred to the expertise of the ECB and the uncertainty as to the consequences of a decision against the OMT program (Kreuder-Sonnen 2019, 149; see also Joerges 2016).^6^

Another issue is that the idea of an emergency constitution does not square well with the nature of EU emergency politics. As White has made clear, what could be observed in the EU context is not a sovereign state of exception as classically conceived, that is, a temporary suspension of certain constitutional provisions and the extension of executive powers, to be revoked once the relevant threat has been tackled, but a phenomenon both more elusive and more far-reaching (White 2015).^7^ During the Euro crisis we saw the creation of new institutions, some of an informal nature (e.g., Troika, Eurogroup), others of a formal kind (e.g., European Stability Mechanism, Fiscal Compact), almost all of which have acquired a permanent character. In other words, EU emergency politics is a mode of *propelling* European integration (Scicluna 2018). The crux of the matter is that institution-building in contravention of established rules and procedures is not something that could be legitimated constitutionally. An emergency constitution may provide a legal basis for narrowly defined measures such as imposing a curfew or deploying the military, while also imposing constraints on executive action, but to use it to provide governments with a practically open-ended mandate for institution-building would contradict the whole point of such an instrument. Finally, there looms the unsolved question of how an emergency constitution could be established in a democratic manner, which leads me to the next strategy.

*Constructive re-appropriation*: A second option is to respond to the results of EU emergency politics with democratic constitution-making. Even if forms of public authority established in crisis periods have a questionable origin and form, it may be possible to embed them in a democratic order ex post. A practical example of this approach is the T-Dem initiative, which calls for new democratic institutions to be built around the EU's economic and social regime as it emerged during the Euro crisis (Hennette et al. 2019; see also Crum and Merlo 2020). What makes this approach attractive is the idea that unconventional adaptations in the EU political system, which may not always be preventable, can be tolerated in crisis situations as long as citizens are able to later re-appropriate the newly created rules or institutions by democratic means.

Again, this approach faces a number of challenges. One obvious difficulty is that EU treaty change requires unanimous consent—the absence of which was exactly one of the conditions that incentivized the contravention of established rules and procedures in the Euro crisis. New institutional structures were set up outside the framework of EU law in order to circumvent the veto of certain member states (de Witte 2013). If unanimity is difficult to achieve under the acute pressure of a crisis, it is all the more so once things have returned to normal—and especially with regard to steps of democratization, as these almost inevitably require a deepening of European integration and the willingness of member state governments and EU executive agencies to give up, or at least share, previously acquired powers.

This leads me to the main problem. If constructive re-appropriation is meant to be a democratic response to emergency politics, it presupposes the capacity of citizens to exercise constituent power—something notoriously lacking in the EU (Patberg 2020a). The current struggles about the Conference on the Future of Europe (the democratic initiative *du jour* at the time of writing in April 2021) once again demonstrate how difficult it is to initiate a truly democratic process of EU reform. The Conference was announced by the new European Commission after the European elections in 2019 as “a bottom-up forum, accessible to all citizens” and meant to give “Europeans a greater say on what the Union does and how it works for them” (European Commission 2020, 1). While this raised the prospect of constitutional change, the interinstitutional debate between European Parliament, Commission, and Council about the Conference mandate quickly made clear that key actors would not allow the forum to become the driver of far-reaching reforms—leading to considerable discontent on the part of civil-society actors (Alemanno et al. 2020). Especially the Council position, according to which the Conference shall “not fall within the scope of Article 48” of the Treaty on European Union, which regulates treaty change, illustrates that democratic constitution-making is not an overly promising response to emergency politics as long as citizens are not in a position to initiate EU reforms against the will of the public authorities whose structure and competences are at stake (Council of the European Union 2020, 7).

*Targeted disintegration*: A third option is to try to reverse the results of EU emergency politics. If it turns out that legal-institutional innovations that have been brought about in a mode of emergency politics lack public support or produce unacceptable consequences, such as a permanent disempowerment of legislatures, or a problematic empowerment of technocratic bodies, the adequate democratic response may be to undo them.

As we are used to associating disintegration with the political rhetoric of Eurosceptics and the uncontrolled decay of hard-won and altogether beneficial forms of international cooperation, this approach requires a few more words of defence than the others. Generally speaking, democracy is nonteleological and always involves the right to do things otherwise. Thus, targeted disintegration can not only be a legitimate response to forms of “runaway integration,” but also has inherent democratic credentials (cf. Patberg 2020b, 587–8). Moreover, it is possible to be both committed to the goal of an “ever closer union” and to pursue limited and deliberate reversals of European integration in order to strengthen the (legitimacy of the) overall process. Crucially, reversal here does not have to amount to a return to the status quo ante, thus completely eliminating the problem-solving capacities or political solutions created in a crisis, but can be limited to particular dysfunctional or normatively problematic (aspects of certain) rules and institutions.

In different ways, both books discussed here point toward the idea of a *democratic politics of disintegration*. Kreuder-Sonnen's case studies show that there is a tendency toward the normalization of emergency measures and in this way indicate that a key democratic capacity needed in the age of inter- and supranational integration is the power of reversal. The difficult question of course is how citizens may be enabled to engage in targeted disintegration. Here, White's idea of principled disobedience could come into play (White 2019, 152–166). Using the example of Syriza in Greece, White argues that party-led forms of intrainstitutional disobedience may be a way to challenge problematic forms of integration enacted in a mode of emergency politics. Through intermediary actors such as party movements, citizens could engage in a form of resistance that is more difficult to ignore than street-level protest and other forms of contestation in the public sphere.

One does not need to point to the failure of Syriza in achieving its political goals in order to see that this approach is also limited in significant ways. I leave aside challenges such as political mobilization and instead focus on a more abstract point. Even if the proposed kind of disobedience could be carried out in an effective and legitimate manner (I would not rule this out), it cannot be more than a preparatory stage for a more constructive form of democratic politics—which it can try to force. If emergency politics has led to the establishment of new rules and institutions, a rollback will usually require treaty change, which means that the question of how citizens could be enabled to exercise constituent power in the EU arises again. In the end, targeted disintegration faces similar problems as constructive re-appropriation. The main advantage it has is that it may sometimes be easier to converge on the goal of reversing certain aspects of European integration than to agree on a positive vision for its future course.

All in all, then, the picture is quite sobering. The possibilities for responding to EU emergency politics by democratic means appear rather limited. Neither regulation in advance nor retroactive containment or reversal are particularly promising strategies. One may doubt, however, whether this has primarily to do with the nature of emergency politics. There is much to suggest that the problem is a more general one—namely, the fact that citizens are generally sidelined in decisions about the shape of the EU constitutional order.

## Global Emergency Politics: Future Trajectories in Politics and Scholarship



Christian Kreuder-Sonnen

Friedrich Schiller University Jena

Jonathan White

The London School of Economics and Political Science

When we first started to write on the transnational politics of emergency, the events of the early 2010s were foremost in our minds. The legacy of the 2008 financial crisis was handled in the EU as a crisis of public debt and eurozone functionality, giving rise to a wave of discretionary measures under the leadership of the Troika. The Euro crisis would become a highly instructive example of how transnational institutions can become entangled in irregular forms of rule. Today though it appears as just one instance among many of the transnational politics of emergency. By the mid-2010s, cross-border migration would be governed in like fashion (Davitti 2018), while from spring 2020 the Covid-19 pandemic would take center-stage (White 2021), also giving renewed significance to the SARS epidemic of 2003 (Hanrieder and Kreuder-Sonnen 2014). In addition, declarations of a *climate* emergency have steadily grown, suggesting further iterations of this mode of politics are yet to come. Here, we take the opportunity both to reflect on the issues raised in this Forum and to highlight areas for future study.

To begin with, while we are convinced that the globalization of risks and crises is very likely to produce further instances of emergency politics beyond the state, less clear is what this means for IOs and global governance. On the one hand, transboundary crises have facilitated “authority leaps” by IOs in several instances, including the WHO during the SARS crisis and the UN Security Council after 9/11 (Heupel, this Forum). Here, emergency politics had a decidedly supranational twist and led to the sustained empowerment of IOs (Kreuder-Sonnen 2019). Yet some apparently similar crises spanning the globe have not had the same effect, with several factors inhibiting cooperation and undermining the authority of IOs. As demonstrated by the so-called migration crisis in Europe, as well as the first months of the coronavirus crisis, globalized emergency politics can also be detrimental to global governance. One important avenue for future research is thus the conditions under which transnational emergency politics either empowers or disempowers international institutions, and with what implications for their legitimacy (Schmidt 2021). One might even say that the present moment is a decisive juncture for international politics and global order per se. In a future likely to be characterized by problems of self-reinforcing interdependence (Hale et al. 2013) and a wave of human-inflicted crises of the Anthropocene, globalized forms of emergency politics are almost certain to come back. Whether they will push global order toward deepened integration or “back” to more Hobbesian international politics is a crucial question, as is that of how to contain the powers arising.

For even if tomorrow's crises lead to increased reliance on international institutions, the one thing our works on emergency politics highlight above all else is that global governance by emergency carries as many problems for democracy and constitutionalism as the domestic state of exception, even if different in form. Koenig-Archibugi (this Forum) helps to pinpoint where the democratic challenges of globalized exceptionalism lie. Separating the *demos* and the *kratos* question, he argues that it is less national governments’ unresponsiveness to their constituents’ demands which is the problem, but rather the clash of interests between nationally conceived demoi in a transnationally integrated polity. In this reading, strengthening the responsiveness of executives toward their citizens might actually exacerbate the issue, at least at the European level. While this highlights an important and underappreciated point about the democratic consequences of transnational emergency politics, we would insist that the *kratos* dimension is more problematic than Koenig-Archibugi suggests. For one, responsiveness is not just about policies in line with opinion polls—it requires deliberation, the possibility to develop dissent and opposition, and it requires transparent procedures. Emergency politics at the European as well as the global level leaves these aspects in short supply (see also Schmidt 2020). Moreover, a focus on the relationship between citizens and their national governments underplays the transnational dimension of emergency politics. Crucial here is that authority is exercised both supranationally and intergovernmentally by more powerful over less powerful states (Kreuder-Sonnen 2019; White 2019). To use the Euro crisis example: the Greek government may have tried to be responsive to the interests of the Greek people, but the democratic problem was that the country was factually under foreign emergency rule by the Troika and Germany. These vertical and “diagonal” aspects of the *kratos* question deserve further exploration.

Heupel's intervention hints at an additional democratic problem, namely that of constituent power. Her piece develops a recurrent theme in debates on the state of emergency more generally, namely the long-term normalization of initially temporary powers. Where emergency politics implies additional authority transfers that become permanent, we are in the realm of constitutional politics in which democratic demands are particularly high (Patberg 2018)—and particularly undermined by practices of executive usurpation. This problem may be especially acute in international institutions, where self-reinforcing veto points and unanimity requirements often make it harder to reverse measures than instituting them in the first place (Scharpf 1988). If we are not to accept as inevitable the global ordering through executive self-empowerment beyond democratic control (with all its normative and sociological consequences), it is important to ask how such tendencies can be checked.

One way to contain emergency politics beyond the state would involve challenging policymakers on their use of justifications of last resort, highlighting political choice against alleged necessity (Séville, this Forum). While we support the thrust of this argument, especially against the purposeful construction or exploitation of crises, one cannot exclude that there will be transboundary situations in which even the best-intentioned crisis managers feel compelled to resort to emergency politics. The Covid-19 pandemic may be a case in point. Séville highlights that the German emergency regime has remained largely within the bounds of the constitution. While it did suspend individual rights and democratic procedures, it did so mainly by following predefined legal steps. At the IO level, such emergency constitutions (Ackerman 2004) rarely exist, and where they do, they are poorly designed (Kreuder-Sonnen 2019). It is thus all the more important to ask if well-designed emergency constitutions could address the normative concerns identified. Regarding the EU, Patberg (this Forum) is sceptical, arguing that an emergency constitution would be unenforceable by the CJEU and unable to constrain the crisis-induced institution-building the EU is so prone to. The more optimistic take would be that it is precisely the conditions for effective judicial review and a limitation of permanent institution-building that a well-designed emergency constitution would (need to) achieve. Admittedly, the idea remains plagued by problems of conception and implementation, but a debate on pre-emptive emergency regulation—in the EU as well as in other international settings—looks warranted (see Kreuder-Sonnen 2021).

How far though should such powers ultimately be constrained—are there not situations that demand an exceptional response, where executives should be free to get on with it? An enduring question in the study of emergency politics is where to strike the balance between constructivist and realist perspectives. Many theorists of the state of exception—notably Schmitt, and Machiavelli and Rousseau before him—have taken a strictly realist view, treating emergencies as given and suggesting the unequivocal need for an exceptional response. Covid-19 ostensibly gives backing to this view: though the virus does not threaten the destruction of a political order (the prime concern for classical theorists), it threatens widespread mortality if ignored. Policy-makers seem to face unarguable constraints.

Yet as Steffek notes, our own books emphasize that emergency politics always involves choices about how to govern and what to prioritize. It is always more than a response to sheer necessity, always a political rather than technical matter. As others have shown, even “natural” disasters associated with droughts, extreme weather or earthquakes produce responses shaped by the social context in which they occur and the political agendas of those involved (see e.g., Keen 2008 on the Sudanese famines of the 1980s, or Klein 2008 on Hurricane Katrina). Among the international actors that govern humanitarian crises, including NGOs, the post-Cold-War period saw the advent of an “emergency imaginary” that encouraged cross-border interventions in the name of stabilizing an exceptional situation (Calhoun 2004). To cast this as a constructivist perspective against a realist one may be to raise a false dichotomy: emergency politics is always the encounter between material challenges and human values, and is therefore never purely real or constructed. If western modernity is prone to crises (Séville, this Forum), how these are handled will always involve a more or less open contest between competing priorities, just as Covid-19 has seen authorities grapple with value choices regarding public health and the economy. This is why we see procedural, including democratic, constraints as crucial. But how far to accept or take distance from the emergency claims of actors will always be an important question.

Emergency rule has often been theorized by its critics. For every Schmittian who aims to defend or celebrate the decisive executive, one finds several constitutional lawyers wanting to rein it in. This has been especially true in the post-War period, where critics of executive discretion have tended to align themselves with the existing liberal order. Is there then a liberal bias to this framework, as Steffek (this Forum) wants to identify in our own work, or a status-quo bias that predisposes one to view new forms of authority sceptically? What can our perspective offer to those of a critical, transformative outlook—to radical democrats, socialists or greens for instance, for whom activist executive agency may be thought crucial? Plenty, we would argue. Rather than study executive exceptionalism as what threatens a nearly just order, one can study it as what inhibits more radical transformations and excludes broader democratic participation. As some of our work on the EU makes clear, transnational emergency politics in recent years has itself often been status-quo oriented—one of the ways in which established interests seek to prop up a capitalist order under pressure. Certainly, there is a risk when analyzing irregular forms of politics that one slips into a conservative orientation, mistrustful of departures from the order of the day, or too willing to see order where there was only flux to begin with. But there is nothing intrinsic to the framework about this, and these are tendencies we aim to avoid in our work.

Looking ahead to how global emergency politics may unfold in the years to come, it is not difficult to see the future as grim. Escalating recourse to exceptional measures seems destined to be paired with escalating opposition, by no means all of it progressive. Anti-lockdown, anti-mask, and anti-vaccination movements have flourished in the wake of Covid-19—all forms of anti-emergency politics, defined by their critique of extreme measures in the name of necessity (White 2021).

Yet it would be wrong to see the future as wholly bleak, and there are important counter-tendencies to the leading patterns we describe. Just as the Coronavirus has been the occasion for governing authorities across the world to embark on emergency rule, it has been the occasion for populations to press them to act in ways they might not have wished. As some governments—for example, in Britain, the United States, and the Netherlands—toyed in spring 2020 with policies intended only to minimize economic disruption and reassure the markets, they met with strong calls to act urgently for the sake of other things—public health, the availability of food, and the wellbeing of ordinary workers. One might think of this as a form of *emergency politics from below* (White 2019). Instead of shoring up the established structures of power and expertise in the manner of executive exceptionalism, this counter-politics of emergency seeks to dispute existing priorities and interests—showing for instance that the demands of an abstraction called “the economy” do not always have to come first. Tackling climate change is going to need this kind of resetting of priorities. Before the Coronavirus, it was uncertain whether political institutions could ever be made responsive to concerns that clash with corporate economic priorities. It is at least now clear that there is a dispute to be had, even in adverse conditions. Recent events give an indication of the global conflicts to come concerning what counts as an emergency and what should be done.

## Footnotes

1 This does not exclude that one finds states of exception in countries other than consolidated liberal democracies. Egypt, for example, provides us with a case in which public authorities derive unfettered coercion from a context of emergency. I thank a reviewer for this clarification.

2 Of course, sometimes they may be drawn to the opposite strategy of denying or trivializing a crisis. Donald Trump's reaction to the COVID-19 pandemic, for instance, was to downplay the scale of the health crisis. He sought to appear heroic and stronger, especially having fought the virus himself, thus signalling that the virus posed no great threat or emergency—that it could be managed by a robust and masculine leader.

3 See also comparative data showing Germany's nonviolation of democratic norms in this period: https://www.v-dem.net/en/analysis/PanDem/.

4 For an authoritative overview: https://yougov.co.uk/topics/international/articles-reports/2020/03/17/perception-government-handling-covid-19.

5 For instance, the Council has refrained from punishing states for not submitting implementation reports for years (UN Security Council 2008, 2), even though such reports would have been due 6 months after the resolution's adoption.

6 A similar argument is made by White, although with emphasis on the relative indeterminacy of EU rules as a possible reason for the restraint of the CJEU (White 2019, 151–152).

7 On Schmitt's notion of a sovereign state of exception and its re-conceptualization for the transnational context, see Steffek's contribution to this Forum.
